# Variable Thickness Strain Pre-Extrapolation for the Inverse Finite Element Method

**DOI:** 10.3390/s23031733

**Published:** 2023-02-03

**Authors:** Dario Poloni, Daniele Oboe, Claudio Sbarufatti, Marco Giglio

**Affiliations:** Mechanical Engineering Department, Politecnico di Milano, Via La Masa 1, 20156 Milano, Italy

**Keywords:** inverse Finite Element Method, iFEM, shape sensing, composite materials, CFRP, GFRP, SHM

## Abstract

The inverse Finite Element Method (iFEM) has recently gained much popularity within the Structural Health Monitoring (SHM) field since, given sparse strain measurements, it reconstructs the displacement field of any beam or shell structure independently of the external loading conditions and of the material properties. However, in principle, the iFEM requires a triaxial strain measurement for each inverse finite element, which is seldom feasible in practical applications due to both costs and cabling-related limitations. To alleviate this problem several techniques to pre-extrapolate the measured strains have been developed, so that interpolated or extrapolated strain values are inputted to elements without physical sensors: the benefit is that the required number of sensors can be reduced. Nevertheless, whenever the monitored components comprise regions of different thicknesses, each region of constant thickness must be extrapolated separately, due to thickness-induced discontinuities in the strain field. This is the case in many practical applications, especially those concerning fiber-reinforced composite laminates. This paper proposes to extrapolate the measured strain field in a thickness-normalized space, where the thickness-induced trends are removed; this novel method can significantly decrease the number of required sensors, effectively reducing the costs of iFEM-based SHM systems. The method is validated in a simple but informative numerical case study, highlighting the potentialities and benefits of the proposed approach for more complex application scenarios.

## 1. Introduction

In past decades, the research community and the mechanical, civil, and aerospace industries have invested significantly in Structural Health Monitoring (SHM). SHM can be regarded as a collection of methodologies [1], sensors [2,3,4], and algorithms [5,6,7,8] that aim to automatically and continuously assess the structural integrity of high-value structures and systems [9,10]. The objective is to lessen and ultimately avoid costly and frequent maintenance inspections on safety-critical assets such as bridges, aircrafts, and vessels, effectively reducing their life cycle cost, increasing their operational lifespan, and improving their overall safety [11,12].

SHM techniques aim to infer the state of health of a structure given measured data from sensors; such techniques may be clustered in data-based black-box models, white-box or physics-based models, and mixed data and physics-based models, also known as grey-box models [13,14]. Black-box methodologies generally make use of Machine Learning techniques to extract damage-sensitive features from the signals’ pattern, with no information on the underlying physics of the problem at hand. In contrast, white-box models make use of physics to interpret signal patterns and infer the health state of structures. Grey-box models combine purely data-based techniques with physics-based ones [13,14].

The inverse Finite Element Method [15,16] makes use of inverse elements to discretize a physical model of a structure; it has proven to be a feasible option to extract structural health indicators, thus recently garnering interest in the SHM field. The iFEM is an inverse shape-sensing [17] method that, given measured strains at discrete locations on the structure, reconstructs the displacement field of any structure that may be modeled as a shell or beam [18], minimizing a weighted functional of the error between the measured strains and their numerical formulation [15]. The inverse Finite Elements are based on shape functions that are not derived from physical equations but from polynomial, approximating kinematic, theories, and the functional minimization is not based on any physical principles; however, the physical model of the structure is still embedded in the iFEM; thus, it may be regarded as a white-box approach in the context of SHM. The most appealing feature of the iFEM is that the external load applied to the structure and material properties are not needed; the geometry and the boundary conditions applied to the structure are the only required parameters [15].

The iFEM has been successfully applied to the monitoring of a container ship [19,20], wind turbines [21,22], and to complex aeronautical structures [23]. Colombo et al. in [24] developed an iFEM-based damage detection methodology, and current research is expanding towards damage identification and quantification [25,26,27,28]; very recent developments are in the direction of a non-deterministic displacement reconstruction [29].

The main drawback of the iFEM is that, for thin shells, in principle the iFEM requires a triaxial strain measurement for each element of the structure, on both the top and bottom of the shell, making it impractical and unsuited for many application scenarios. To relieve this issue, two approaches were introduced that would reduce the number of required sensors for the iFEM. The first one can be called weighting, it was introduced in the very first iFEM formulation [15]. The second one is the use of strain interpolation or extrapolation techniques, such as the Smoothing Element Analysis (SEA) [30,31,32], which have been introduced only more recently [33,34].

Weighting means assigning a lower weight in the iFEM functional to the elements that do not comprise any sensor in their spatial domain, or to the strain components that are not measured, such as the transverse shear strain, whose contribution to the displacement is generally negligible in thin shells. Although weighting has generally a positive effect on displacement reconstruction, its effect is limited, and it does not arbitrarily affect the solution [35].

Strain pre-extrapolation techniques interpolate or extrapolate the measurements on the top and bottom of the shell in the elements missing experimental measurements before the iFEM computes the displacement field; this solution has been proven to increase the accuracy of the iFEM methods and to reduce the number of sensors required to achieve a prescribed performance [33,34,36]. Any interpolation/extrapolation method may be employed in principle; the state-of-the-art employs the SEA, modal expansion [37], and physics-based pre-extrapolation techniques [38], although polynomial fittings may be robust enough for simpler case studies. It should be noted that the pre-extrapolated values do not have to be exactly equal to the true values that would be measured by sensors since weights may alleviate this discrepancy [33,34], and the displacement field reconstructed by the iFEM restores the compatibility in the displacement field. The interested reader may refer to [33] for a comparison of the SEA and polynomial fittings as strain pre-extrapolation techniques, while the SEA and modal expansion methods are explored in [37].

The main issue with extrapolating the strain measurements on both the top and bottom of the shell is that it is inefficient for application scenarios where the component thickness is not constant, which is typical of fiber-reinforced composite components and stiffened components [36]. In fact, the strain field depends on the component thickness, and severe discontinuities in the strain field are present due to the variations in the thickness itself. As will be shown in the case study, even for simple cases, such as a simple cantilever plate of piecewise constant thickness subjected to pure bending, the state of the art requires each region of constant thickness to be extrapolated separately, thus requiring at least some strain sensors (according to the specific interpolation technique adopted) for any interpolation region.

This paper proposes an alternative approach to extrapolate the strain field so that the main discontinuities induced by the change in thickness are removed. The key idea is to scale the membrane strain and the curvatures depending on the component thickness, since in any isotropic material and in the great majority of composite layup sequences used in engineering applications the laminate membrane stiffness scales with the thickness, and the bending stiffness scales with the third power of the thickness. Thus, by multiplying the membrane strains by the laminate thickness and the curvatures by the third power of the thickness, the normalized membrane strains and the normalized curvatures can be pre-extrapolated considering measurements placed on regions of different thickness, since this normalization removes the discontinuities in the strain field induced by the variations in thickness. Following the pre-extrapolation, the normalized membrane strains and curvatures can be unnormalized and fed as input to the iFEM.

The main benefit of the proposed approach is that it makes a global pre-extrapolation feasible for variable thickness components, instead of resorting to multiple local pre-extrapolations as in the state of the art: this translates into a decrease in the number of required sensors, thus in a significant reduction of the costs and complexity of iFEM-based SHM systems.

The effectiveness of the proposed approach is demonstrated in a numerical experiment, which considers a cantilever plate subjected to different loading conditions: albeit simple, this case study is particularly informative, highlighting the potentialities and the benefits of the proposed approach for more complex application scenarios.

The remainder of the paper is structured as follows. Section 2 provides a brief introduction to the inverse Finite Element Method and describes the variable thickness pre-extrapolation methodology. Section 3 presents the case study and comments on the results. Section 4 concludes the work underlying future research directions.

## 2. The Inverse Finite Element Method

A brief introduction to the inverse FEM is presented in this section, while a more comprehensive and detailed overview is available in [15,24,34] for the interested reader.

The iFEM minimizes a least square functional of the error between measured strains and curvatures and their respective numerical formulations to compute the displacement field of any shell or beam structure discretized into finite elements. The parameters required for the method are the structural geometry and the boundary conditions.

The iFEM formulation employed in this paper makes use of the iQS4 elements [39] for plates and shells, which is based on the Mindlin kinematic assumptions and whose shape functions were originally developed for the MIN4 element [40,41]. For each inverse Finite Element i the membrane strains $e_{i}$, the curvatures $k_{i}$ and the transverse shear strains $g_{i}$ are measured ($\cdot^{\epsilon }$) (see Section 2.2 for a more detailed explanation of the sensors and measurements), and their respective numerical formulation. $\left(u^{i}\right)$ is computed as a function of the nodal degrees of freedom $u^{i}$; the error least-square functional $\Phi_{i}$ is built as:

(1) $$\Phi_{i}\left(u^{i}\right)=w_{m}^{i}\|e\left(u^{i}\right)-e_{i}^{\varepsilon }{\|}^{2}+w_{b}^{i}\|k\left(u^{i}\right)-k_{i}^{\varepsilon }{\|}^{2}+w_{s}^{i}\|g\left(u^{i}\right)-g_{i}^{\varepsilon }{\|}^{2}$$

where the coefficients $w_{m}^{i}$, $w_{b}^{i}$, and $w_{s}^{i}$ are positive weights associated with the related strain components for each element. The role of the weights is twofold: first, it controls the coherence between the strain field components; secondly, it can weight the contribution of different elements that are linked to the same nodal degrees of freedom.

Controlling the coherence is desirable in thin shell applications, where the transverse shear strains g cannot be measured directly: given that their contribution to the displacement is negligible in thin shells, they are set to zero, and the weight $w_{s}^{i}$ is set to a value of $10^{-3}$ for all the elements, so that more importance is given to the minimization of the error of the membrane and bending components. 

The weights are also used to compensate for the fact that experimental measurements are typically not available for all the elements: In this case, the measurements ($\cdot^{\epsilon }$) are set to zero, or they are pre-extrapolated from the sensor data [33,34], and the value of the weight is reduced to a value ranging from $10^{-1}$ and $10^{-3}$, to take into account the fact that the input value is not a direct experimental measurement. It should be noted that altering the weights does not arbitrarily change the solution, but it can improve the solution up to a limit [35]. Although not implemented since it is out of the scope of this work, the weights might also be assigned a different value for each strain direction, as detailed in [42].

The next two subsections describe how the numerical formulation of the strains ($\cdot^{\epsilon }$) and their experimental counterpart ($\cdot^{\epsilon }$) are obtained, while Section 2.3 describes the Euclidean norms in Equation (1) and the functional minimization.

### 2.1. Numerical Strain Formulation

**Figure 1:**
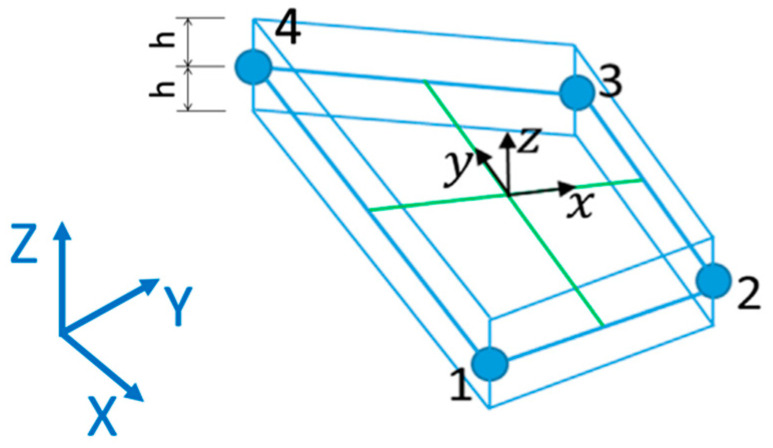
Local (x, y, z) and global (X, Y, Z) reference systems for the iQS4 element; numbers 1 to 4 represent the local node labels.

The numerical formulation of the strains ($e\left(u^{i}\right),\;g\left(u^{i}\right)$) and of the curvatures $k\left(u^{i}\right)$ is derived from the bilinear shape functions originally developed for the MIN4 element [40,41]. They are defined in a local reference system (x, y, z) whose origin is set in the centroid of the inverse finite element, as illustrated in Figure 1; the local coordinate spans the element thickness, with z∈[−h;+h], where h is the element semi-thickness. The iQS4 element has 4 nodes, with three translational and three rotational degrees of freedom per node, for a total of 24 degrees of freedom, which are stacked in the vector $u^{i}$. The degrees of freedom of the nodes $u^{i}$ in local coordinates are linked to the strain by means of the derivatives of the shape functions, namely $B^{m}$, $B^{b}$, $B^{g}$ (membrane, bending and shear) in the following equation. Their formulation is not reported here for the sake of brevity, but the interested reader can refer to [39] where they are explicitly detailed.

(2) $$e\left(u^{i}\right)=B^{m}u^{i}\;k\left(u^{i}\right)=B^{b}u^{i}\;g\left(u^{i}\right)=B^{s}u^{i}$$

### 2.2. Input Strain Formulation

**Figure 2:**
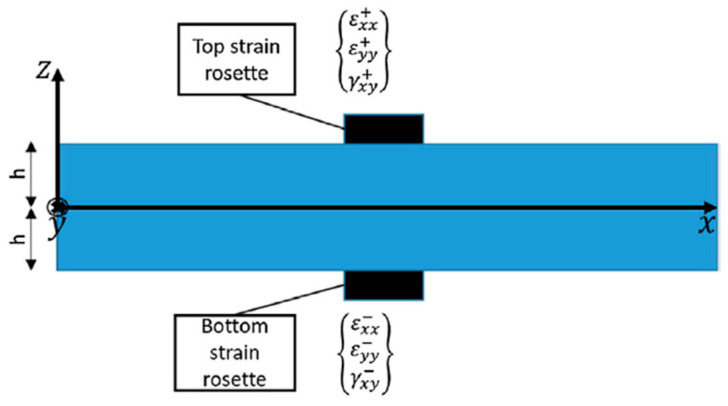
Discrete sensor location on the shell structure.

Each inverse Finite Element in principle requires at least a measurement of the membrane strains $e_{i}^{\varepsilon }$ and of the curvatures $k_{i}^{\varepsilon }$. Given that the sensors (typically strain gauge rosettes) are applied on the top and bottom of the shell, as illustrated in Figure 2, the membrane strain and the curvatures with respect to the element mid-plane are recovered by making use of the Mindlin kinematic assumptions, as reported in the following equation, where $\varepsilon_{xx}$, $\varepsilon_{yy}$ denote respectively the measurement of the strain in the x and y direction, while $\gamma_{xy}$ denote the shear strain component.

(3) $$e_{i,j}^{\varepsilon }={\left\{\begin{array}{c} \varepsilon_{xx}^{0}\\ \varepsilon_{yy}^{0}\\ \gamma_{xy}^{0} \end{array}\right\}}_{j}=\frac{1}{2}{\left\{\begin{array}{c} \varepsilon_{xx}^{+}+\varepsilon_{xx}^{-}\\ \varepsilon_{yy}^{+}+\varepsilon_{yy}^{-}\\ \gamma_{xy}^{+}+\gamma_{xy}^{-} \end{array}\right\}}_{j}\;\;\;\;\;\;\;\;\;\;\;\;\;\;\;\;\;\;\;\;k_{i,j}^{\varepsilon }={\left\{\begin{array}{c} \kappa_{xx}^{0}\\ \kappa_{yy}^{0}\\ \kappa_{xy}^{0} \end{array}\right\}}_{j}=\frac{1}{2h}{\left\{\begin{array}{c} \varepsilon_{xx}^{+}-\varepsilon_{xx}^{-}\\ \varepsilon_{yy}^{+}-\varepsilon_{yy}^{-}\\ \gamma_{xy}^{+}-\gamma_{xy}^{-} \end{array}\right\}}_{j}$$

The subscript j in the equation denotes the fact that there may be more sensors for each Finite Element, although this is rarely the case in practice; the element thickness 2h is computed for each sensor location, and the superscripts $\left(\cdot^{+}\right)$ and $\left(\cdot^{-}\right)$ denote that the measurements are taken, respectively, at the top and bottom of the shell. As mentioned, the transverse shear g cannot be recovered from measurements taken on the shell surface. Nevertheless, its contribution is negligible in thin shells, and as it is common practice in the iFEM, it will be disregarded from here on.

### 2.3. Euclidean Norms and Minimization

To minimize the global error functional, the Euclidean norms defined in Equation (1) are expanded as in the following equation, where it is assumed that n strain sensors are embedded into the *i*-th element.

(4) $$\|e\left(u^{i}\right)-e_{i}^{\varepsilon }{\|}^{2}=\frac{1}{n}\iint_{A_{i}}^{}\overset{n}{\underset{j=i}{\sum }}{\left(e{\left(u^{i}\right)}_{j}-e_{i,j}^{\varepsilon }\right)}^{2}dxdy\;\|k\left(u^{i}\right)-k_{i}^{\varepsilon }{\|}^{2}=\frac{{\left(2h\right)}^{2}}{n}\iint_{A_{i}}^{}\overset{n}{\underset{j=i}{\sum }}{\left(k{\left(u^{i}\right)}_{j}-k_{i,j}^{\varepsilon }\right)}^{2}dxdy\;\|g\left(u^{i}\right)-g_{i}^{\varepsilon }{\|}^{2}=\frac{1}{n}\iint_{A_{i}}^{}\overset{n}{\underset{j=i}{\sum }}{\left(g{\left(u^{i}\right)}_{j}-g_{i,j}^{\varepsilon }\right)}^{2}dxdy$$

where $A_{i}$ is the element area. It should be noted that whenever no input data is available for a particular strain component, i.e., it has not been pre-extrapolated, n is conventionally set to 1 and the strain component is set to zero: a small weighting coefficient is then applied, as previously described.

By making use of Equations (2) and (3), the least-square functional of the *i*-th inverse element presented in Equation (1) can be reduced to the following equation:

(5) $$\Phi_{i}\left(u^{i}\right)=u^{i^{T}}K^{i}u^{i}-2u^{i^{T}}f^{i}+\xi^{i}$$

where:

(6) $$k^{i}=\iint_{A_{i}}^{}\left(w_{m}B^{m^{T}}B^{m}+w_{b}{\left(2h\right)}^{2}B^{b^{T}}B^{b}+w_{s}B^{s^{T}}B^{s}\right)dxdy\;f^{i}=\frac{1}{n}\iint_{A_{i}}^{}\overset{n}{\underset{j=1}{\sum }}\left(w_{m}B^{m^{T}}e_{i,j}^{\varepsilon }+w_{b}{\left(2h\right)}^{2}B^{b^{T}}k_{i,j}^{\varepsilon }+w_{s}B^{s^{T}}g_{i,j}^{\varepsilon }\right)dxdy\;\xi^{i}=\frac{1}{n}\iint_{A_{i}}^{}\overset{n}{\underset{j=1}{\sum }}\left(w_{m}e_{i,j}^{\varepsilon^{T}}e_{i,j}^{\varepsilon }+w_{b}{\left(2h\right)}^{2}k_{i,j}^{\varepsilon^{T}}k_{i,j}^{\varepsilon }+w_{s}g_{i,j}^{\varepsilon^{T}}g_{i,j}^{\varepsilon }\right)dxdy$$

The global functional can be built by resorting to a standard assembly operation, in which the displacements in the local coordinates are transformed into global coordinates. The boundary conditions are applied so that rigid-body motion is avoided, and the minimization (∂Φ/∂U=0) is carried out analytically, yielding the following linear problem:

(7) $$U_{F}=K_{FF}^{-1}\cdot F_{F}$$

where $U_{F}$ is the vector of global displacements, $K_{FF}$ is a matrix resembling the stiffness matrix in the direct FEM, and $F_{F}$ is a forcing term that corresponds to the contribution of the input strains. It should be noted from the above equations that the matrix $K_{FF}$ does not depend on the input strains: once it is inverted, the method can be applied in real-time by updating the forcing vector $F_{F}$, which is a computationally inexpensive operation since it only involves matrix multiplications.

### 2.4. Variable Thickness Pre-Extrapolation with the iFEM

As mentioned above, in practical applications, it is usually not feasible to install one sensor per each inverse Finite Element, and pre-extrapolation techniques are employed to interpolate and extrapolate the measured strains to the locations of the inverse elements which are not covered by any sensor.

To understand the idea that drives the proposed approach, it is helpful to briefly recap the generalized constitutive law of plates according to the classical laminate theory; the interested reader can refer to [43] for a more detailed illustration. For a laminate composed of r layers, the stresses in the *l*-th layer can be expressed as a function of the membrane strains and curvatures by transforming the lamina stiffness matrix ${\bar{Q}}^{l}$ from the lamina axis to the laminate axis $\left(\overset{=}{Q}\right)$, yielding:

(8) $${\left[\begin{array}{c} \sigma_{xx}\\ \sigma_{yy}\\ \sigma_{xy} \end{array}\right]}^{l}={\left[\begin{array}{ccc} {\overset{=}{Q}}_{11} & {\overset{=}{Q}}_{12} & {\overset{=}{Q}}_{16}\\ {\overset{=}{Q}}_{21} & {\overset{=}{Q}}_{22} & {\overset{=}{Q}}_{26}\\ {\overset{=}{Q}}_{16} & {\overset{=}{Q}}_{26} & {\overset{=}{Q}}_{66} \end{array}\right]}^{l}\left(\left[\begin{array}{c} \begin{array}{c} \varepsilon_{xx}^{0}\\ \varepsilon_{yy}^{0}\\ \varepsilon_{xy}^{0} \end{array} \end{array}\right]+z\left[\begin{array}{c} \begin{array}{c} \kappa_{xx}^{0}\\ \kappa_{yy}^{0}\\ \kappa_{xy}^{0} \end{array} \end{array}\right]\right)={\overset{=}{Q}}^{l}\left(e+zk\right)$$

The resultant forces N and moments M per unit length are by definition:

(9) $$N=\left[\begin{array}{c} N_{xx}\\ N_{yy}\\ N_{xy} \end{array}\right]=\left[\begin{array}{c} \int_{-h}^{h}\sigma_{xx}\;dz\\ \int_{-h}^{h}\sigma_{yy}\;dz\\ \int_{-h}^{h}\sigma_{xy}\;dz \end{array}\right]=\overset{r}{\underset{l=1}{\sum }}{\overset{=}{Q}}^{l}\int_{-z_{l-1}}^{z_{l}}\left(e+zk\right)\;dz\;M=\left[\begin{array}{c} M_{xx}\\ M_{yy}\\ M_{xy} \end{array}\right]=\left[\begin{array}{c} \int_{-h}^{h}\sigma_{xx}\;z\;dz\\ \int_{-h}^{h}\sigma_{yy}\;z\;dz\\ \int_{-h}^{h}\sigma_{xy}\;z\;dz \end{array}\right]=\overset{r}{\underset{l=1}{\sum }}{\overset{=}{Q}}^{l}\int_{-z_{l-1}}^{z_{l}}\left(ez+z^{2}k\right)\;dz$$

where $z_{l-1}$ and $z_{l}$ are, respectively, the lower and upper out-of-plane coordinates of each lamina, and $\sigma_{\cdot }$ denotes the Cauchy stress tensor components. Given a stacking sequence, the well-known symmetric relationship can be derived:

(10) $$\left[\begin{array}{c} N\\ M \end{array}]=[\begin{array}{cccccc} A_{11} & A_{12} & A_{16} & B_{11} & B_{12} & B_{16}\\ A_{12} & A_{22} & A_{26} & B_{12} & B_{22} & B_{26}\\ A_{16} & A_{26} & A_{66} & B_{16} & B_{26} & B_{66}\\ B_{11} & B_{12} & B_{16} & D_{11} & D_{12} & D_{16}\\ B_{12} & B_{22} & B_{26} & D_{12} & D_{22} & D_{26}\\ B_{16} & B_{26} & B_{66} & D_{16} & D_{26} & D_{66} \end{array}][\begin{array}{c} \varepsilon_{xx}^{0}\\ \varepsilon_{yy}^{0}\\ 2\varepsilon_{xy}^{0}\\ \kappa_{xx}^{0}\\ \kappa_{yy}^{0}\\ \kappa_{xy}^{0} \end{array}]=[\begin{array}{cc} A & B\\ B & D \end{array}][\begin{array}{c} e\\ k \end{array}\right]$$

where matrix A governs the membrane laminate stiffness, matrix B couples the membrane and bending components of the laminate, and D is the bending stiffness matrix. 

The present work restricts the application of the proposed methodology to the application scenarios where there is no membrane-bending coupling, and matrix B is null. This is typically not an issue given that symmetric lamination sequences, for which matrix B is null and there is no membrane-bending coupling, are employed in the vast majority of fiber-reinforced polymer applications due to manufacturing issues with non-symmetric laminates. Whenever B is not null, the laminate twists or bends after the curing process, since it naturally shrinks when it is cooled from the curing temperature to the ambient temperature: this change of curvature is generally deleterious; therefore, it is avoided by design in most cases, since it makes it particularly difficult to predict the shape of the cured component.

The proposed methodology further restricts its scope to the materials where the membrane matrix A scales with the thickness of the laminate, and the bending matrix D scales with the third power of the thickness of the laminate 2h. Albeit this restriction may appear a severe limitation, it is almost irrelevant in practice given that most of the fiber reinforced polymers practical applications employ homogeneous stacking sequences, as thoroughly detailed in [44]: these sequences satisfy this constraint.

For the sake of clarity, the analytical expression of the constitutive law is reported for an isotropic material in the next equation.

(11) $$[\begin{array}{c} N_{xx}\\ N_{yy}\\ N_{xy}\\ M_{xx}\\ M_{yy}\\ M_{xy} \end{array}]=\left[\begin{array}{cccccc} \frac{E\left(2h\right)}{1-v^{2}} & v\frac{E\left(2h\right)}{1-v^{2}} & 0 & 0 & 0 & 0\\ .. & \frac{E\left(2h\right)}{1-v^{2}} & 0 & 0 & 0 & 0\\ .. & .. & \frac{E\left(2h\right)}{1+v} & 0 & 0 & 0\\ .. & .. & .. & \frac{E{\left(2h\right)}^{3}}{12\left(1-v^{2}\right)} & v\frac{E{\left(2h\right)}^{3}}{1-v^{2}} & 0\\ .. & .. & .. & .. & \frac{E{\left(2h\right)}^{3}}{12\left(1-v^{2}\right)} & 0\\ .. & .. & .. & .. & .. & \frac{E{\left(2h\right)}^{3}}{12\left(1+v\right)} \end{array}][\begin{array}{c} \varepsilon_{xx}^{0}\\ \varepsilon_{yy}^{0}\\ \gamma_{xy}^{0}\\ \kappa_{xx}^{0}\\ \kappa_{yy}^{0}\\ \kappa_{xy}^{0} \end{array}\right]$$

where E is the Young Modulus of the material and ν the Poisson ratio.

Given that the membrane stiffness matrix A and the bending stiffness matrix D are, respectively, proportional to h and $h^{3}$, we propose a novel pre-extrapolation approach that normalizes the membrane strain and the curvatures by multiplying membrane strains for h and the curvatures for $h^{3}$, as per the following equation:

(12) $$\eta =\left\{\begin{array}{c} \eta_{xx}\\ \eta_{yy}\\ \eta_{xy} \end{array}\right\}=e\cdot \;h\;\xi =\left\{\begin{array}{c} \xi_{xx}\\ \xi_{yy}\\ \xi_{xy} \end{array}\right\}=k\cdot h^{3}$$

where η will be called normalized membrane strains and ξ normalized curvatures. For the sake of clarity, the pre-extrapolation procedure is described in Algorithm 1.
**Algorithm 1**: Variable Thickness Pre-Extrapolation1:Given surface measurements, compute curvatures k and membrane strains e (Equation (3)).2:Normalize the k and e, computing the normalized curvatures ξ and membrane strains η (Equation (9)).3:Pre-extrapolate/interpolate the normalized membrane strain components using any interpolation/extrapolation method.4:Unnormalize ξ and η (Equation (12)) to recompute k and e**.**5:k and e to the iFEM input.

The reason why pre-extrapolating in the normalized space is beneficial is that it removes most of the discontinuities in the strain field induced by thickness changes, as is shown in the next section. The normalization, albeit not equivalent, under the aforementioned assumptions yields the same effect as if the internal forces and moments were pre-extrapolated, rather than the strain field itself. It should be noted that, although the fact that the laminate material properties must be thickness-homogeneous and there must not be any membrane-bending coupling in the laminate constitutive law, the actual values of the material properties are not needed, and no material calibration must be performed to apply the proposed method.

The proposed approach should preferably be applied to thin laminates, which are employed in most practical aeronautical thin-walled structures. Thick laminates should be handled with care, as the proposed approach is applicable provided that the Mindlin kinematic assumptions are acceptable: This depends on the transverse shear stiffness of the particular laminate that is considered. The proposed approach might be applicable for stiffened plates with slight modifications, as long as the homogeneity of the layup is preserved in the stiffener base, and the framework hereby proposed is modified by considering possible variations in the mean plane of the shell: the validation for stiffened plates is left for future research. Sandwich structures are generally of uniform thickness, as their main weakness and least efficient constituent is the core; therefore, the applications with variable thickness are rather scarce. However, as for thick laminates, sandwich structures must be modelled with Timoshenko’s kinematic theory, which implies that both bending and transverse shear stiffness contribute to structural displacement. Therefore, the proposed approach is not applicable in the current form to such structures, and this extension might be developed in future research.

## 3. Case Study: Composite Variable Thickness Plate

The proposed methodology is validated in a simple but informative numerical case study: a composite plate made of fiberglass and epoxy resin clamped at one tip and subjected to different loading conditions. The procedure is as follows: a direct FEM analysis is run, and the displacement and strain fields are computed as output. The strain field at the sensor locations is extracted from the direct FEM analysis, as if it were measured by sensors; it is pre-extrapolated and given as input to the iFEM: The iFEM displacement field is compared to the direct FEM analysis, which is assumed to be the ground truth. No sensor noise has been simulated, given that the purpose of this case study is to highlight the potentiality of the method without any confounding influence.

**Figure 3:**
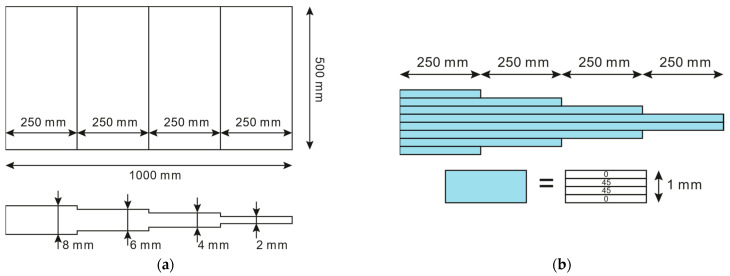
(**a**) Composite plate dimensions and (**b**) layup sequence.

**Figure 4:**
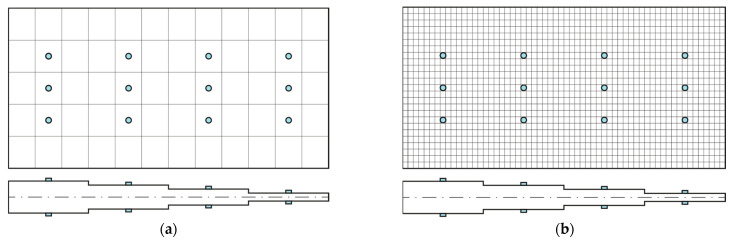
(**a**) iFEM mesh and (**b**) direct FEM mesh: Triaxial strain sensors are placed on both the bottom and top sides at each blue-filled circle.

The plate dimensions and layup sequence are depicted in Figure 3. Each lamina is composed of a fiberglass weave and epoxy resin, which is widely used in wind turbine applications. The lamina properties are reported in Table 1. The direct FEM and the iFEM meshes are shown in Figure 4, along with the sensor network: Sensors are placed on both the top and bottom sides of the plate, as is required by the iFEM. The membrane $w_{m}^{i}$ and bending $w_{b}^{i}$ weights assigned to the elements where the solution is pre-extrapolated, i.e., all the elements which do not contain any sensor in Figure 4a, are set to $10^{-1}$, while the weights for the transverse shear strain $w_{s}^{i}$ are set to $10^{-3}$ as is common practice in the iFEM literature, given that they are not measured and their contribution to the displacement is negligible in thin shells. No sensitivity analysis on the values of the weights has been performed since, as empirically shown in [35]; whenever the pre-extrapolated strains are close to the true strain, as in this case study, the sensitivity to the weights is negligible.

In this case study polynomials are used to pre-extrapolate the strain field, although in principle any pre-extrapolation technique may be applicable. This is because every reader should be familiar with polynomial pre-extrapolation, while more complex strain extrapolation techniques, such as the SEA, would have not been more effective and would have shifted attention away from the objective of the analysis, which is to evaluate the effectiveness of the normalization.

The loading conditions applied to the plate are traction, out-of-plane tip load, uniform pressure, and shear loading: They have been chosen since each of them induces a specific deformation mode either in the membrane strain or in the curvature components, so that it is possible to promptly perceive the effects of the proposed pre-extrapolation methodology. The following subsections present the results of the iFEM analysis for each load case.

### 3.1. Traction

**Figure 5:**
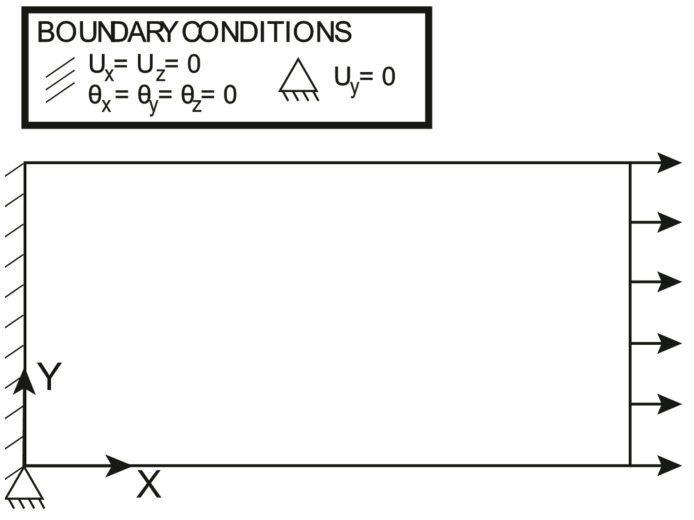
Traction: loading and boundary conditions.

The boundary conditions and the loading conditions applied to the plate are shown in Figure 5: the plate is clamped at one end and the uniformly distributed line load applied on the other end is set to $15\frac{N}{\mathrm{mm}}$. It should be noted that the transverse displacement $U_{y}$ is constrained just in one point at the clamped edge to avoid any boundary effect due to the clamp, which is induced by the Poisson ratio; this is to focus attention just on the effectiveness of the proposed approach to deal with variable thickness, rather than focusing on the local effects of the boundary conditions. The polynomial pre-extrapolation orders for the normalized membrane strains and the normalized curvatures are reported in Table 2: It should be noted that the only pre-extrapolation of interest is $\eta_{\mathrm{XX}}$, as the traction force induces a pure membrane loading. The polynomial pre-extrapolation degree for $\eta_{\mathrm{XX}}$ is set to 1 for the X direction, while it is set to 2 for the Y direction because it achieves a slightly better fit with respect to a bilinear fit. For the sake of completeness, the authors have run an iFEM simulation with a bilinear pre-extrapolation, and the results are almost identical to the ones hereby reported.

**Figure 6:**
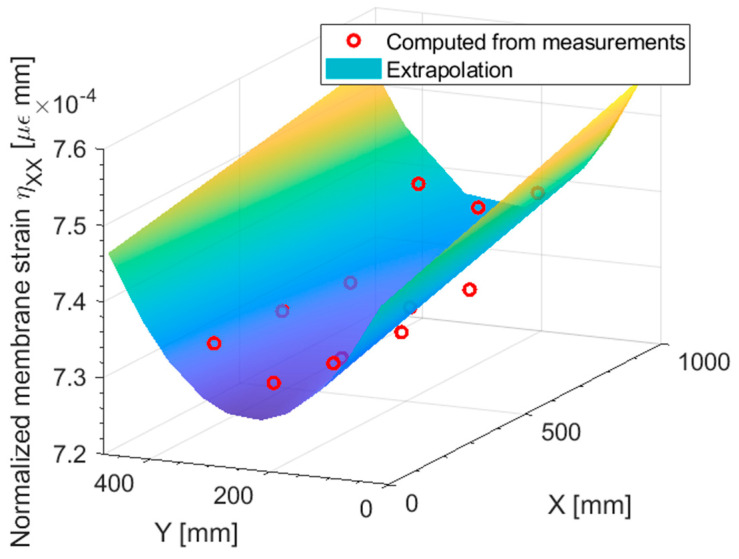
Traction: normalized membrane strain $\eta_{XX}$ pre-extrapolation.

**Figure 7:**
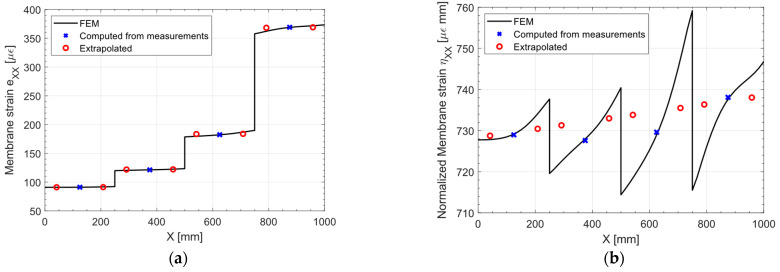
Traction: (**a**) membrane strain $e_{XX}$ and (**b**) normalized membrane strain $\eta_{XX}$, slice at Y = 250 mm.

Figure 6 presents the normalized membrane strains $\eta_{\mathrm{XX}}$ computed from the measurements on both the top and bottom surfaces of the shell, along with the pre-extrapolation. At a glance, the first order polynomial should be a good fit for the normalized strain. To better appreciate the goodness of fit of this pre-extrapolation in a two-dimensional plot, Figure 7 displays a slice at Y = 250 (half the plate width) of both the normal strain $e_{XX}$ and the normalized normal strains $\eta_{XX}$: The true strain distribution given by the direct FEM analysis is overlayed for comparison.

Looking at the direct FEM distribution in the membrane strain, Figure 7a, two general trends may be noticed: a major trend with harsh discontinuities, induced by the variable thickness in the specimen, and some minor trends on the portions of the plate of constant thickness. It is apparent that by looking at the $e_{XX}$ at the measurements (blue cross marks in Figure 7a), the only feasible approach to correctly pre-extrapolate the membrane strain would be to use a constant function for each portion of the plate with constant thickness. However, this approach is not feasible when the strain field is not piecewise constant, as it will be shown in the next sections, and whenever there is a region of constant thickness which do not contain any sensor.

On the other hand, looking at the normalized plot presented in Figure 7b, it is apparent that the major discontinuities induced by the variable thickness are removed, and the direct FEM presents only residual trends. These residual trends, given the sparsity of the sensor network, are not captured, but the linear fit (red circles in Figure 7b) is reasonable given the measured points (blue cross marks in Figure 7b). This is confirmed by the unnormalized pre-extrapolated membrane strain displayed in Figure 7a: the pre-extrapolated values are indeed close to the direct FEM, demonstrating the goodness of the pre-extrapolation methodology.

**Figure 8:**
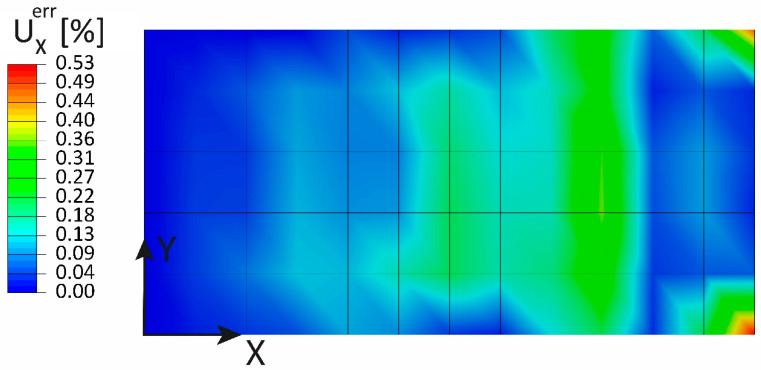
Traction: percentage error of the $U_{X}$ displacement.

The other strain and curvature components pre-extrapolation plots are not reported for the sake of brevity, given that their contribution to the displacement component of interest is negligible for this load case. The relative error between the displacement $U_{X}$ computed by the direct FEM and the one computed by the iFEM is shown in Figure 8: the maximum relative error is 0.53%, and the error peaks are located at the two corners of the specimen.

### 3.2. Out-of-Plane Tip Loading

**Figure 9:**
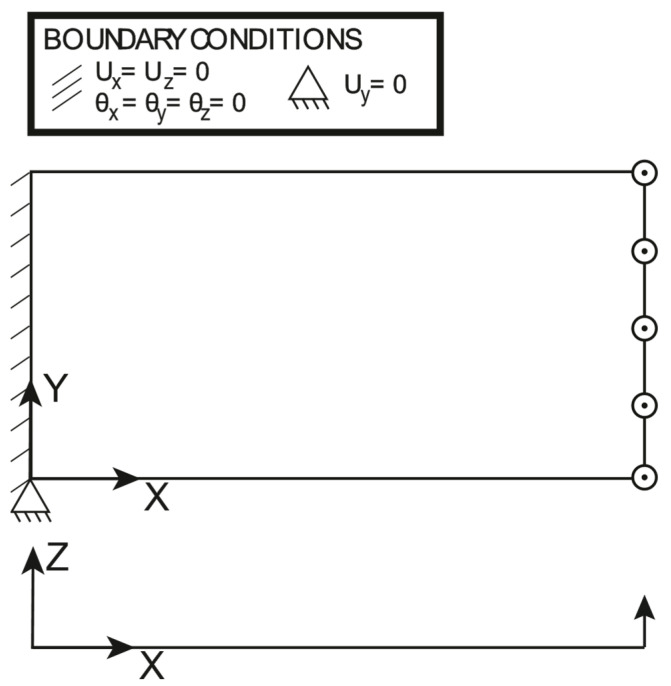
Out-of-plane tip loading: loading and boundary conditions.

The boundary and loading condition of the plate for this load case are presented in Figure 9: the out of plane line load has been set to $0.01\;\frac{N}{\mathrm{mm}}$, achieving an out-of-plane displacement of 12 mm at the plate loaded end in the direct FEM analysis, which is consistent with the assumption of linearity in the displacements both in the direct and inverse FEM. The polynomial pre-extrapolation degrees for this load case are reported in Table 3; the most relevant component to pre-extrapolate to achieve a good displacement reconstruction is $\xi_{XX}$, i.e., the normalized curvature in the longitudinal direction: the polynomial degree has been set to one, given that for a scenario with constant thickness, the curvature should be linear, as the bending moment. Although all the other pre-extrapolation components have been set to 1, they are actually irrelevant for this load case, but they are reported for the sake of completeness.

**Figure 10:**
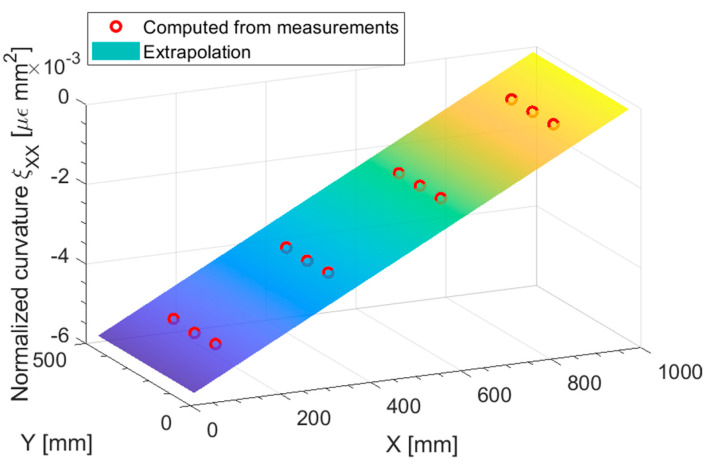
Out-of-plane tip loading: normalized curvature $\xi_{XX}$ pre-extrapolation.

**Figure 11:**
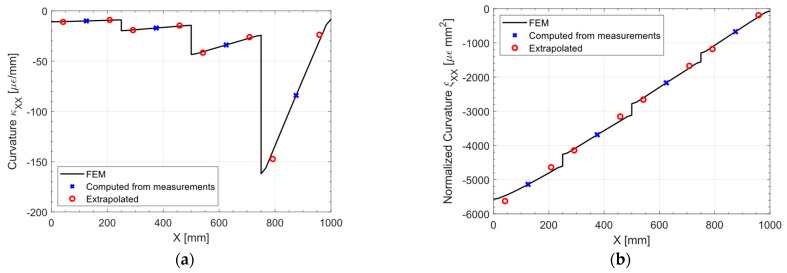
(**a**) out-of-plane tip loading: curvature $\kappa_{XX}$ and (**b**) normalized curvature $\xi_{XX}$, slice at Y = 250 mm.

Figure 10 shows the pre-extrapolated curvature and the one computed from the measurements: the bilinear pre-extrapolation achieves a perfect fit of the data. In Figure 11 the pre-extrapolation methodology can be appreciated: from Figure 11a it is apparent that the direct-FEM curvature exhibits a piecewise linear trend, which would be impossible to extrapolate given the sensor network sparsity. The only solution to capture this trend would be to place at least two sensors for each portion of the plate of constant thickness, as the state of the art would suggest. By normalizing the curvature as in Figure 11b, the discontinuities and the slope changes induced by the variable thickness are suppressed: the linear fit is both in agreement with the observed data and the direct FEM solution. The minor discontinuities in the direct FEM normalized curvature presented in Figure 11b are almost negligible given the fact that they are in the normalized space: once the unnormalized pre-extrapolated values are remarkably close to the direct FEM solution.

**Figure 12:**
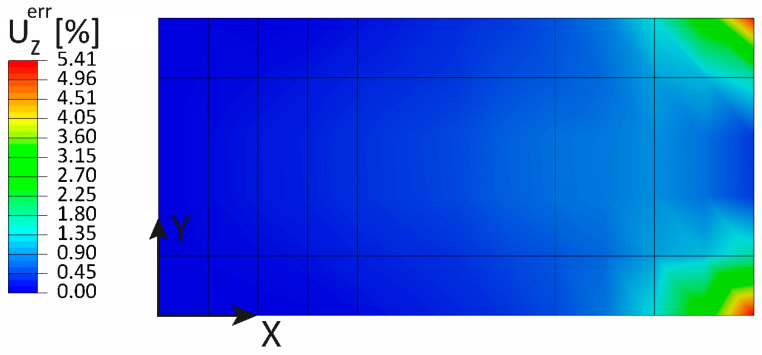
Out-of-plane tip loading: percentage error of the $U_{Z}$ displacement.

The relative error between the iFEM-reconstructed displacement $U_{Z}$ and the direct FEM is shown in Figure 12: the maximum percentage error is 5.41%, located at the two corners of the free end, while it tends to zero in the central region of the loading edge, highlighting the goodness of the pre-extrapolation adopted.

### 3.3. Uniform Pressure

**Figure 13:**
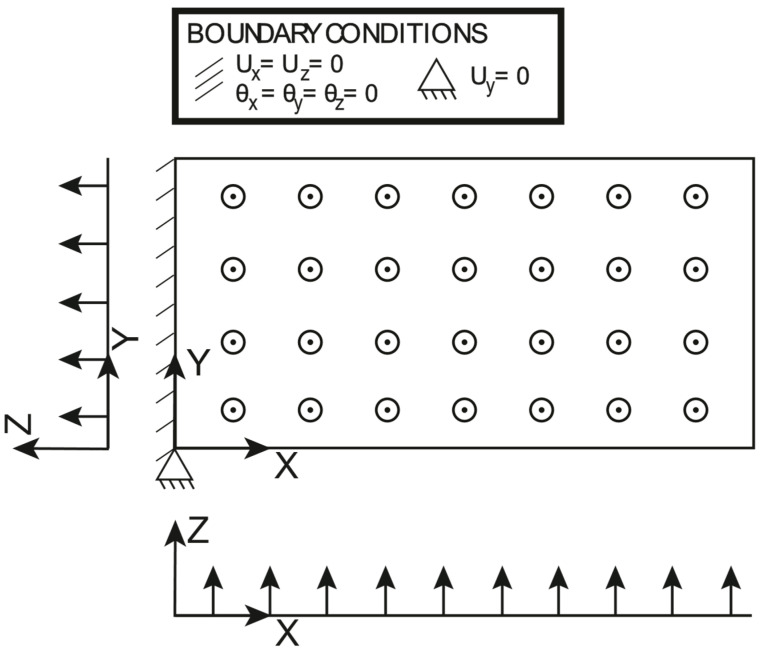
Uniform pressure: loading and boundary conditions.

**Figure 14:**
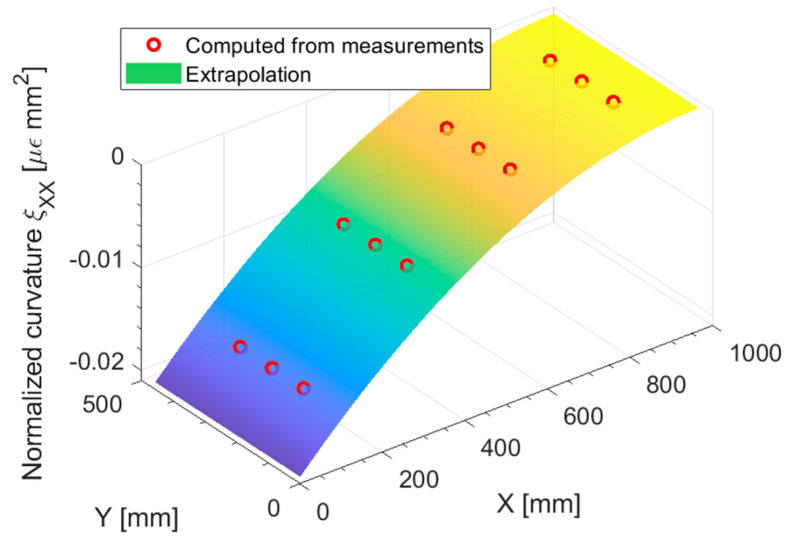
Uniform pressure: normalized curvature $\xi_{XX}$ pre-extrapolation.

In this load case, the plate is subjected to a uniform pressure distribution; for the sake of clarity, the boundary and loading conditions are illustrated in Figure 13. The pressure magnitude is set to $7.5\cdot 10^{-5}\frac{N}{{\mathrm{mm}}^{2}}$, and the maximum displacement given by the direct FEM is 21.8 mm, so that the hypothesis of linearity is considered acceptable. Table 4 reports the degrees of the polynomial used to pre-extrapolate and interpolate the data: a quadratic polynomial is used to fit the only component of interest, i.e., $\kappa_{XX}$, in the X direction, given that the data follow a parabolic trend in the normalized space. The polynomial fit for $\kappa_{XX}\;$is shown in Figure 14.

**Figure 15:**
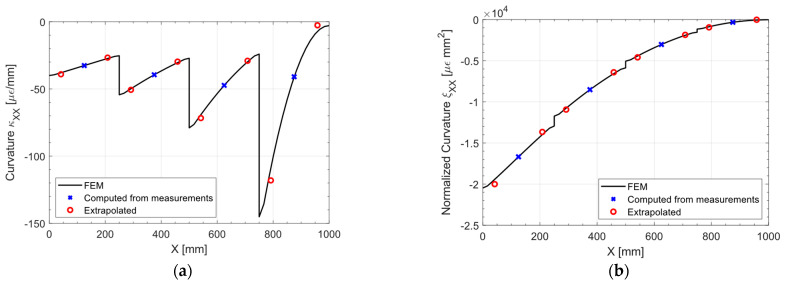
Uniform pressure: (**a**) curvature $\kappa_{XX}$ and (**b**) normalized curvature $\xi_{XX}$, slice at Y = 250 mm.

The curvature $\kappa_{XX}$ is shown in Figure 15a: the solution is piecewise parabolic, and the state of the art would require at least two sensing points per each portion of the plate of constant thickness to achieve a piecewise linear fit, three sensing points if a piecewise quadratic function is desired. By normalizing the curvature $\kappa_{XX}$ as in Figure 15b, the piecewise quadratic $\kappa_{XX}$ is transformed into an almost quadratic function, which can be interpolated by fewer sensors. As in the previous load cases, some minor trends in the normalized strain $\xi_{XX}$ are persistent and are not removed by the normalization. Notwithstanding, the pre-extrapolated strain $\kappa_{XX}$ in Figure 15a is deemed a fair approximation of the direct FEM curvature. For this and the previous load cases, it should be noted that, in principle, there is no need for all the regions of constant thickness to be covered by sensors and potentially one or more regions might not include any sensor without compromising the displacement reconstruction if the normalized strain field can be assumed sufficiently smooth, i.e., some a priori knowledge of the loading conditions is available, as in any practical application.

**Figure 16:**
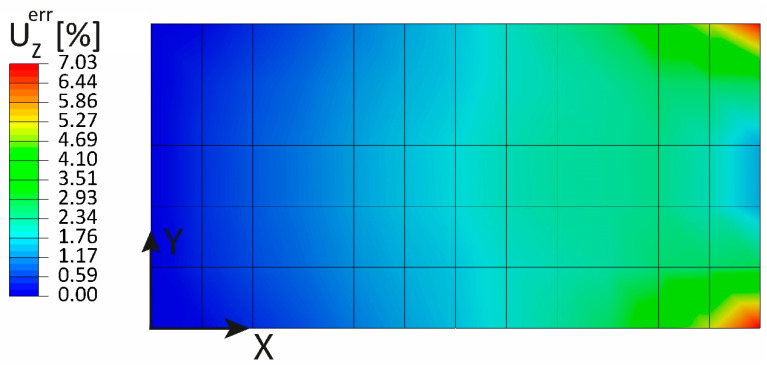
Uniform pressure: percentage error of the $U_{Z}$ displacement.

The percentage error of the iFEM displacement $U_{Z}$ reconstruction with respect to the direct FEM displacement is shown in Figure 16: The maximum error is 7%, which validates the quality of the pre-extrapolation.

### 3.4. Shear Loading

**Figure 17:**
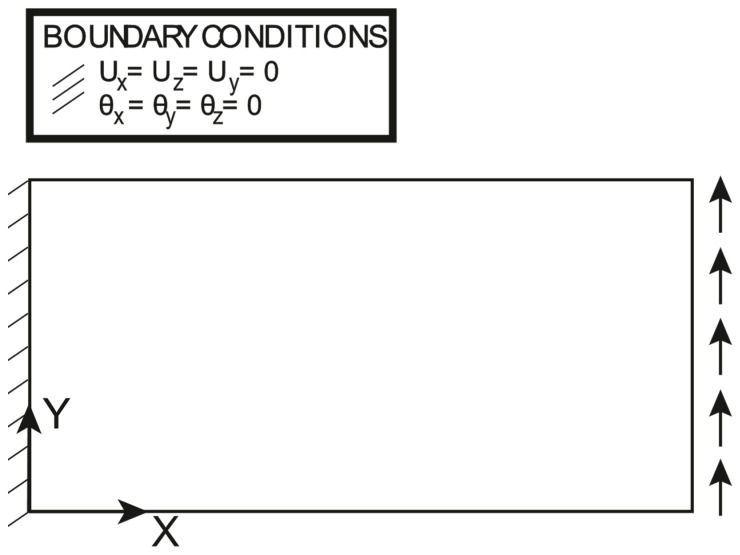
Shear loading: loading and boundary conditions.

The boundary conditions and the loading conditions for this load case are reported in Figure 17: the applied shear load is set to $7.5\;\frac{N}{\mathrm{mm}}$. It should be noted that the clamping condition differs from the one presented in the previous section, and in this case, all the degrees of freedom are constrained at X=0 mm. The polynomial pre-extrapolation setup is reported in Table 5; the strain component of interest for this load case is $\eta_{XY}$: A linear fitting in the X direction is performed, while a quadratic fit is chosen for the Y direction.

**Figure 18:**
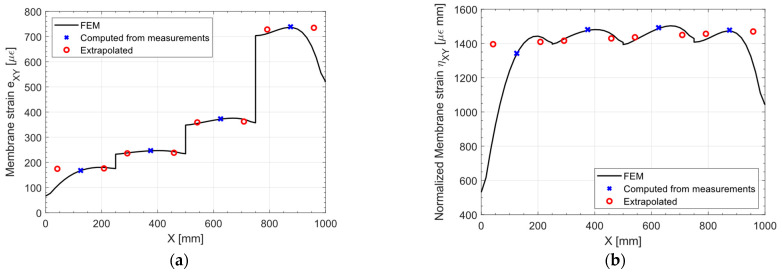
Shear loading: (**a**) membrane strain $e_{XY}$ and (**b**) normalized membrane strain $\eta_{XY}$, slice at Y = 250 mm.

**Figure 19:**
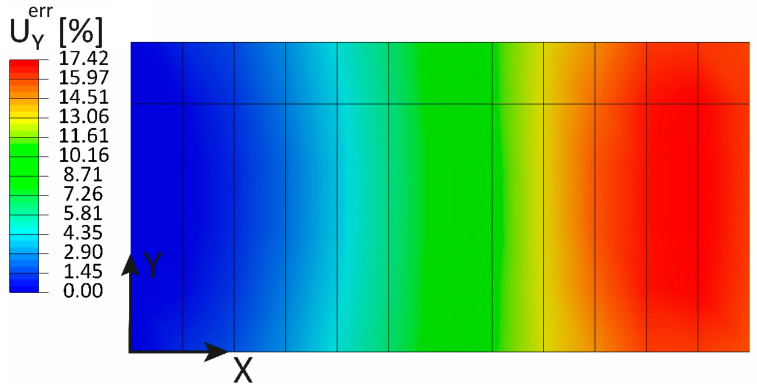
Shear loading: percentage error of the $U_{Y}$ displacement.

The considerations outlined for the previous load cases are still valid for this loading scenario: The discontinuities induced by the variable thickness are removed by the normalization, as shown in Figure 18. However, the shear load and the boundary conditions induce a more complex strain pattern with respect to the previous cases, especially from X=0 mm to X=250 mm and from X=750 mm to X=1000 mm: the membrane strain $e_{XY}$ and its normalized counterpart $\eta_{XY}$ decrease significantly in these sections due to local effects. The sensor network is not able to capture these variations in the strain field, and the extrapolated values differ significantly from the direct FEM. Hence, it comes as no surprise that the maximum percentage error is about 17% as shown in Figure 19. Although the relative error is large, it is not of particular interest since the main objective of this dissertation is to show that the discontinuities in the strain field in the normalized space are successfully removed.

## 4. Conclusions

This work proposes a novel approach to pre-extrapolate and interpolate the strain field for the iFEM so that the global variations in the strain field induced by thickness changes are removed. The proposed methodology is promising since it can significantly reduce the number of required strain sensors needed for the shape-sensing of complex structures of variable thickness, such as airplane wings or wind turbine blades.

The minor drawback of the proposed approach is that the material properties must be partially known: the laminate stiffness properties must be thickness-homogeneous so that the membrane and bending stiffnesses scale with the thickness and the third power of the thickness, respectively, and there must be no membrane-bending coupling in the laminate constitutive law.

The proposed approach has been applied to a simple case study of a clamped plate subjected to several different loading conditions, making use of polynomial pre-extrapolation. Its simplicity not only demonstrates the potential of the method in more complex application scenarios, but also stresses the need for such an approach even in elementary applications.

Future research may be devoted to applying the proposed approach to more complex case studies, such as aircraft structural frames, possibly including an experimental validation. More conceptually, the proposed approach may be expanded by pre-extrapolating the laminate moments and forces, so that any arbitrary constitutive law may be accounted for.

## Figures and Tables

**Table 1:** Fiberglass-epoxy lamina material properties [45] for the direct FEM.

$E_{11}\;$ [MPa]	$E_{22}\;\left[MPa\right]$	$\nu_{12}$	$G_{12}\;\left[MPa\right]$	$G_{13}\;\left[MPa\right]$	$G_{23}\;\left[MPa\right]$	Thickness [mm]
26,000	26,000	0.1	3800	2800	2800	0.25

**Table 2:** Traction: selected polynomial pre-extrapolation orders.

	$\eta_{XX}$		$\eta_{YY}$		$\eta_{XY}$		$\xi_{XX}$		$\xi_{YY}$		$\xi_{XY}$	
Direction	X	Y	X	Y	X	Y	X	Y	X	Y	X	Y
Polynomial degree	1	2	1	1	1	1	1	1	1	1	1	1

**Table 3:** Out-of-plane tip loading: selected polynomial pre-extrapolation orders.

	$\eta_{XX}$		$\eta_{YY}$		$\eta_{XY}$		$\xi_{XX}$		$\xi_{YY}$		$\xi_{XY}$	
Direction	X	Y	X	Y	X		X	Y	X	Y	X	Y
Polynomial degree	1	1	1	1	1	1	1	1	1	1	1	1

**Table 4:** Uniform pressure: selected polynomial pre-extrapolation orders.

	$\eta_{XX}$		$\eta_{YY}$		$\eta_{XY}$		$\xi_{XX}$		$\xi_{YY}$		$\xi_{XY}$	
Direction	X	Y	X	Y	X	Y	X	Y	X	Y	X	Y
Polynomial degree	1	1	1	1	1	1	2	1	1	1	1	1

**Table 5:** Shear loading: selected polynomial pre-extrapolation orders.

	$\eta_{XX}$		$\eta_{YY}$		$\eta_{XY}$		$\xi_{XX}$		$\xi_{YY}$		$\xi_{XY}$	
Direction	X	Y	X	Y	X	Y	X	Y	X	Y	X	Y
Polynomial degree	1	1	1	1	1	2	1	1	1	1	1	1

## Data Availability

Not applicable.
